# Nrf2 signaling activation by a small molecule activator compound 16 inhibits hydrogen peroxide-induced oxidative injury and death in osteoblasts

**DOI:** 10.1038/s41420-022-01146-7

**Published:** 2022-08-08

**Authors:** Jing-wei Zhao, Pei-jun Tang, Zhen-tao Zhou, Gang Xu, Quan Li, Ke-ran Li, Yue-huan Zheng

**Affiliations:** 1 Division of Spine Surgery, Department of Orthopedics, Tongji Hospital, Tongji University School of Medicine, Shanghai, China; 2 Department of Pulmonary, The Affiliated Infectious Hospital of Soochow University, Suzhou, China; 3 Department of Orthopedics, The Second Affiliated Hospital of Soochow University, Suzhou, China; 4 Center of Stomatology, The Second Affiliated Hospital of Soochow University, Suzhou, China; 5 The Fourth School of Clinical Medicine, The Affiliated Eye Hospital, Nanjing Medical University, Nanjing, China; 6 Department of Orthopedics, Ruijin Hospital, Shanghai Jiao Tong University School of Medicine, Shanghai, China

**Keywords:** Stress signalling, Osteoporosis

## Abstract

We explored the potential activity of compound 16 (Cpd16), a novel small molecule Nrf2 activator, in hydrogen peroxide (H_2_O_2_)-stimulated osteoblasts. In the primary murine/human osteoblasts and MC3T3-E1 murine osteoblastic cells, Cpd16 treatment at micro-molar concentrations caused disassociation of Keap1-Nrf2 and Nrf2 cascade activation. Cpd16 induced stabilization of Nrf2 protein and its nuclear translocation, thereby increasing the antioxidant response elements (ARE) reporter activity and Nrf2 response genes transcription in murine and human osteoblasts. Significantly, Cpd16 mitigated oxidative injury in H_2_O_2_-stimulited osteoblasts. H_2_O_2_-provoked apoptosis as well as programmed necrosis in osteoblasts were significantly alleviated by the novel Nrf2 activator. Cpd16-induced Nrf2 activation and osteoblasts protection were stronger than other known Nrf2 activators. Dexamethasone- and nicotine-caused oxidative stress and death in osteoblasts were attenuated by Cpd16 as well. Cpd16-induced osteoblast cytoprotection was abolished by Nrf2 short hairpin RNA or knockout, but was mimicked by Keap1 knockout. Keap1 Cys151S mutation abolished Cpd16-induced Nrf2 cascade activation and osteoblasts protection against H_2_O_2_. Importantly, weekly Cpd16 administration largely ameliorated trabecular bone loss in ovariectomy mice. Together, Cpd16 alleviates H_2_O_2_-induced oxidative stress and death in osteoblasts by activating Nrf2 cascade.

## Introduction

Osteoporosis and osteonecrosis are extremely common systemic bone diseases that are characterized by significantly decreased bone mass as well as progressive architectural deterioration in the bone [1–3]. Studies have implied that excessive reactive oxygen species (ROS) and oxidative cell injury are the major contributors for the development and progression of osteoporosis and osteonecrosis [4–6]. Specifically, ROS can lead to profound oxidative injury to osteoblasts [7–9]. Hydrogen peroxide (H_2_O_2_) and other oxidative stimuli (dexamethasone, nicotine and etc.) are added to osteoblasts/osteoblastic cells [10–16]. These stimuli can led to significant oxidative injury and robust death of osteoblasts [11–14, 16].

Nrf2 drives the transcription of a significant number of antioxidant and cytoprotective genes, through association with a *cis*-acting element (ARE/EpRE) in the promoter region in the nuclei [17–21]. In the resting condition, however, Nrf2 is negatively regulated by Keap1, the latter initiates poly-ubiquitination and degradation machinery to promote Nrf2 protein degradation and stops Nrf2 translocation into cell nuclei [18, 19]. Therefore small molecules that block the Keap1-Nrf2 binding should extent the Nrf2’s half-life, stabilizing Nrf2 protein, causing its accumulation in cytosol and subsequent translocation to nuclei, thereby activating Nrf2 signaling cascade [17–21].

Pharmacological or genetic activation of Nrf2 cascade can efficiently protect osteoblasts/osteoblastic cells against oxidative injury by H_2_O_2_ [10, 13, 15, 16, 22–24] and other oxidative stimuli [25–29]. We have previously shown that four-octyl itaconate (4-OI) activated Nrf2 cascade through alkylating Keap1’s cysteine residues and potently inhibited H_2_O_2_-induced osteoblast death [10]. Moreover, iKeap1, a direct and novel Keap1 inhibitor that was discovered by the structure-based virtual screening, inhibited H_2_O_2_-induced osteoblast death by activating Nrf2 signaling cascade [30]. In addition, MIND4-17, the novel activator that uniquely activated Nrf2 signaling cascade by separating Nrf2-Keap1 complex, protected osteoblasts from H_2_O_2_-induced oxidative injury and death [16]. Chlorogenic acid significantly attenuated H_2_O_2_-caused oxidative stress and death in MC3T3-E1 osteoblastic cells by activating Nrf2 signaling cascade [15].

Genetic strategies were also utilized to activate Nrf2 signaling cascade. microRNA-455 (miR-455) silenced Cullin 3, thereby activating Nrf2 signaling and protecting osteoblasts against oxidative stress [13]. Liang et al. have recently shown that a novel microRNA, microRNA-4523, silenced phosphoglycerate kinase 1 (PGK1) to stimulate Nrf2 signaling, protecting human osteoblasts from dexamethasone-caused oxidative injury [31]. Other Nrf2-activating miRNAs, including miR-200a [28], miR-107 [32], and miR-19a [26], also offered significant osteoblast cytoprotection by suppressing oxidative injury.

Marcotte et al. have recently developed a small molecule inhibitor of Nrf2-Keap1 interaction named compound 16 (Cpd16, PubChem CID 1073725) [33]. Cpd16 binds directly to the Keap1’s Kelch-DC domain at the C-terminus of Keap1 with the IC_50_ of 2.7 µM [33]. It was able to increase Nrf2 response genes in cultured cells and acted as a novel Nrf2 activator [33]. Here our study reported that Cpd16 activated Nrf2 cascade and protected osteoblasts from H_2_O_2_.

## Results

### Cpd16 activates Nrf2 signaling cascade in osteoblasts

**Fig. 1:**
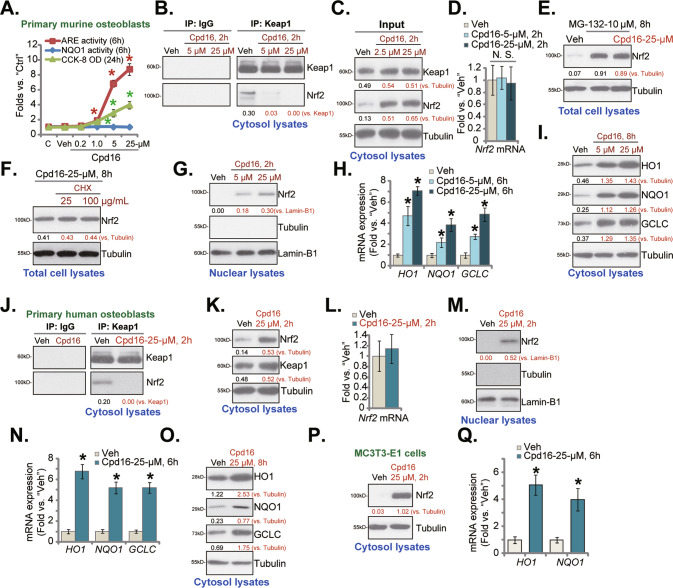
Cpd16 activates Nrf2 signaling cascade in osteoblasts. Primary murine osteoblasts (**A**–**D**, **G**–**I**), human osteoblasts (**J**–**O**), or the MC3T3-E1 murine osteoblastic cells (**P**, **Q**) were stimulated with Cpd16, ARE activity and NQO1 activity as well as cell viability were measured (**A**); Keap1-Nrf2 association was measured through co-immunoprecipitation (Co-IP) (**B**, **J**); Proteins in cytosol/nuclear fraction lysates were examined (**C**, **G**, **I**, **K**, **M**, **O**, **P**), with mRNAs measured by qRT-PCR (**D**, **H**, **L**, **N**, **Q**). The primary murine osteoblasts were treated with MG-132 (10 μM) or plus Cpd16 (25 μM) for 8 h, total protein lysates were tested (**E**). The murine osteoblasts were pretreated for 1 h with cycloheximide (CHX, 25/100 μg/mL), following by Cpd16 (25 μM) stimulation for another 8 h, listed proteins were shown (**F**). “C” is untreated control (same for all Figures). “Veh” is vehicle control (0.1% DMSO) (same for all Figures). *P < 0.05 *versus* “Veh” cells.

As shown, Cpd16 dose-dependently enhanced ARE luciferase reporter activity in murine osteoblasts (Fig. 1A). Further indicating Nrf2 cascade activation, NQO1 enzyme activity was remarkably increased after 1–25 μM of Cpd16 treatment (Fig. 1A). Cpd16 at 0.2 μM failed to significantly increase the NQO1 enzyme activity, showing the dose-dependent response (Fig. 1A). CCK-8 assay results found that Cpd16 (0.2–25 μM, for 24 h) failed to significantly decrease the viability in murine osteoblasts (Fig. 1A), suggesting that the compound is relatively safe to murine osteoblasts. At the two concentrations, 5 μM and 25 μM, Cpd16 robustly increased ARE reporter activity and the NQO1 enzyme activity in murine osteoblasts (Fig. 1A), they were selected for the following experiments.

Treatment with Cpd16 (5 μM and 25 μM for 2 h) disrupted association of Keap1-Nrf2 in primary murine osteoblasts (Fig. 1B). As a result, the cytosol Nrf2 protein levels were significantly increased (Fig. 1C). On the contrary, Cpd16 failed to significantly alter Keap1 protein (Fig. 1C) and *Nrf2* mRNA expression (Fig. 1D) in murine osteoblasts. These results implied that Cpd16 induced Keap1-Nrf2 departure and stabilization of Nrf2 protein in murine osteoblasts. Notably, Cpd16 (25 μM) was unable to further increase Nrf2 protein levels in murine osteoblasts with MG-132 co-treatment (Fig. 1E). Moreover, cycloheximide (CHX), the known protein synthesis inhibitor [34], did not alter Nrf2 protein expression in Cpd16 (25 μM)-treated primary murine osteoblasts (Fig. 1F). These results further supported Nrf2 protein stabilization following Cpd16 treatment in cultured osteoblasts.

Stabilized Nrf2 protein translocated from cytosol to nuclei of the murine osteoblasts (Fig. 1G), which is an initial and key step for activation of the Nrf2 cascade [18, 19, 35]. Indeed, Nrf2-transcripted genes, including *HO1*, *GCLC,* and *NQO1* [10, 16, 28, 30, 36], were remarkably elevated following Cpd16 (5/25 μM) treatment (Fig. 1H). Their protein levels, tested by Western blotting assays, were increased as well (Fig. 1I).

In human osteoblasts [10, 30], 25 μM of Cpd16 treatment disrupted Keap1 immunoprecipitation with Nrf2 (Fig. 1J) as well, leading to stabilization of Nrf2 protein in cytosol (Fig. 1K). Keap1 protein (Fig. 1K) and *Nrf2* mRNA (Fig. 1L) were not significantly changed. After testing the nuclear fraction lysates, we showed that the accumulated Nrf2 protein translocated to the nuclei (Fig. 1M). Consequently, expression of Nrf2 response genes were significantly increased (Fig. 1N, O). Similarly in MC3T3-E1 murine osteoblastic cells, Cpd16 treatment stabilization Nrf2 protein (Fig. 1P) and increased mRNA expression of Nrf2 response genes (Fig. 1Q). Together, Cpd16 activated Nrf2 signaling in osteoblasts.

### Cpd16 ameliorates H_2_O_2_-provoked oxidative injury in osteoblasts

**Fig. 2:**
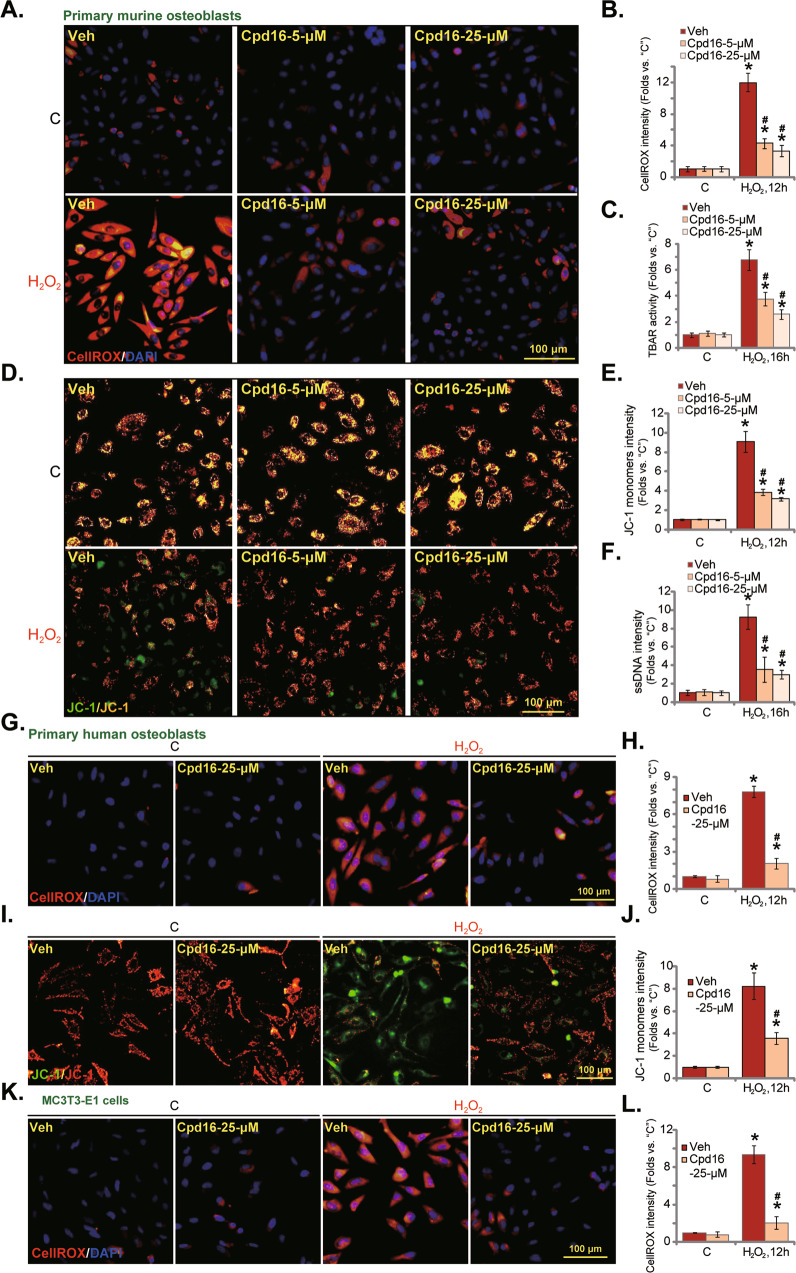
Cpd16 ameliorates H_2_O_2_-provoked oxidative injury in osteoblasts. Primary murine osteoblasts (**A**–**F**), human osteoblasts (**G**–**J**), or the MC3T3-E1 murine osteoblastic cells (**K** and **L**) were pretreated (for 2 h) with Cpd16 (5/25 μM), or plus H_2_O_2_ (400 μM) stimulation; ROS contents (the CellROX intensity assay, **A**, **B**, **G**, **H**, **K**, **L**), TBAR activity (**C**), JC-1 dye staining (**D**, **E**, **I**, **J**), and ssDNA contents (**F**) were measured. *P < 0.05 *versus* “C” cells. ^#^*P* < 0.05 *versus* cells with H_2_O_2_ stimulation but “Veh” pretreatment. Scale bar = 100 μm.

By measuring the CellROX fluorescence intensity, we demonstrated that H_2_O_2_ induced robust ROS production (CellROX intensity increase) in the primary murine osteoblasts (Fig. 2A, B). Significantly, pretreatment with Cpd16 (5/25 μM) potently inhibited H_2_O_2_-induced ROS production (Fig. 2A, B). Moreover, H_2_O_2_-induced lipid peroxidation, or TBAR activity increase, was largely inhibited by Cpd16 pretreatment as well (Fig. 2C). In addition, in murine osteoblasts the novel Nrf2 activator largely attenuated H_2_O_2_-induced mitochondrial depolarization (tested by the formation of JC-1 monomers, Fig. 2D, E). ssDNA accumulation indicated enhanced DNA breaks in H_2_O_2_-treated murine osteoblasts (Fig. 2F), which was suppressed by Cpd16 pretreatment (Fig. 2F).

Pretreatment with Cpd16 (25 μM) in the primary human osteoblasts significantly ameliorated H_2_O_2_-stimulated ROS production (Fig. 2G, H) and mitochondrial depolarization (formation of JC-1 monomer, Fig. 2I, J). In MC3T3-E1 murine osteoblastic cells, pretreatment with Cpd16 also potently inhibited H_2_O_2_-stimulated ROS production (Fig. 2K, L). Thus, Cpd16 ameliorated H_2_O_2_-induced oxidative injury in osteoblasts.

### Cpd16 ameliorates H_2_O_2_-induced apoptosis and programmed necrosis in osteoblasts

**Fig. 3:**
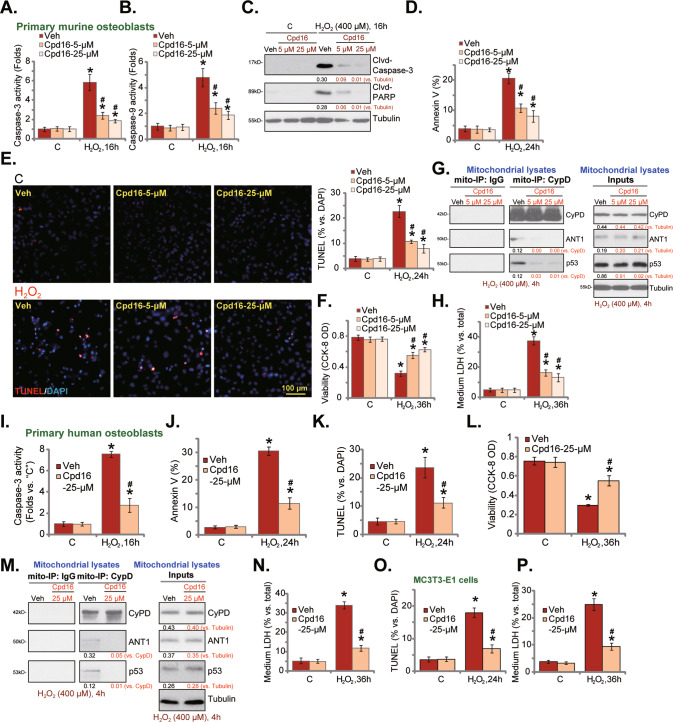
Cpd16 ameliorates H_2_O_2_-induced apoptosis and programmed necrosis in osteoblasts. Primary murine osteoblasts (**A**–**H**), human osteoblasts (**I**–**N**) or the MC3T3-E1 murine osteoblastic cells (**O**, **P**) were pretreated (for 2 h) with Cpd16 (5/25 μM), or plus H_2_O_2_ (400 μM) stimulation; the caspase-3/-9 activities (**A**, **B**, **I**) were measured; apoptosis-associated proteins were measured (**C**); cell apoptosis was examined by Annexin V flow cytometry (**D**, **J**, results were quantified) and the nuclear TUNEL staining (**E**, **K**, and **O**, results were quantified) assays, with cell viability measured through CCK-8 assays (**F**, **L**); CyPD-ANT1-p53 mitochondrial complexation and the expression were shown (**G**, **M**), and cell necrosis measured through measuring LDH releasing (**H**, **N**, **P**). *P < 0.05 *versus* “C” cells. ^#^*P* < 0.05 *versus* cells with H_2_O_2_ stimulation but “Veh” pretreatment. Scale bar = 100 μm.

H_2_O_2_ will cause death of cultured osteoblasts [10, 13, 16, 22, 30, 37, 38]. In line with our previous findings [10, 30], H_2_O_2_ exerted pro-apoptotic activity in cultured murine osteoblasts, as it increased the caspase-3/-9 activities (Fig. 3A, B) and caused caspase-3 and PARP cleavages (Fig. 3C), which were ameliorated by pretreatment of Cpd16 (5/25 μM). H_2_O_2_ provoked apoptosis in murine osteoblasts, increasing Annexin-positive staining cells (Fig. 3D) and TUNEL-nuclei staining (Fig. 3E). Cpd16 potently reduced H_2_O_2_-induced apoptosis activation in murine osteoblasts (Fig. 3D, E). Moreover, H_2_O_2_-induced cytotoxicity or CCK-8 viability reduction (Fig. 3F), was alleviated by Cpd16 as well.

Besides apoptosis, studies have shown that H_2_O_2_ together other oxidative stimuli could simultaneously provoke programmed necrosis cascade [10, 13, 30, 39, 40]. It is a mitochondria-dependent active cell necrosis cascade that can be initiated by mitochondrial CyPD (cyclophilin D)-ANT1 (ADP/ATP translocase 1)-p53 association [41–43]. H_2_O_2_ (400 μM, 4 h) stimulation in murine osteoblasts indeed induced CyPD-ANT1-p53 association in the mitochondria [10, 30], and pretreatment with Cpd16 (5/25 μM) significantly inhibited the complex formation (Fig. 3G). To supporting necrosis induction, medium LDH levels were increased following H_2_O_2_ treatment in murine osteoblasts (Fig. 3H), which was again inhibited by Cpd16 (Fig. 3H). These results supported that the novel Nrf2 activator also inhibited programmed necrosis cascade activation in murine osteoblasts.

In the primary human osteoblasts pretreatment with Cpd16 dramatically inhibited H_2_O_2_-induced caspase-3-apoptosis activation (Fig. 3I–K). Apoptosis induction was evidenced by the quantified results from Annexin-V flow cytometry (Fig. 3J) and TUNEL nuclei staining (Fig. 3K) assays. In addition, pretreatment with Cpd16 (25 μM) inhibited H_2_O_2_-induced viability reduction (Fig. 3L). Furthermore, H_2_O_2_-induced mitochondrial association of CyPD-ANT1-p53 (Fig. 3M) and LDH releasing (Fig. 3N) were significantly inhibited by the novel Nrf2 activator in human osteoblasts. In MC3T3-E1 osteoblastic cells, H_2_O_2_-induced apoptosis (TUNEL assays, Fig. 3O) and necrosis (Fig. 3P) were inhibited by Cpd16 pretreatment as well.

### Cpd16-induced Nrf2 activation and osteoblasts protection against H_2_O_2_ were stronger than other known Nrf2 activators

**Fig. 4:**
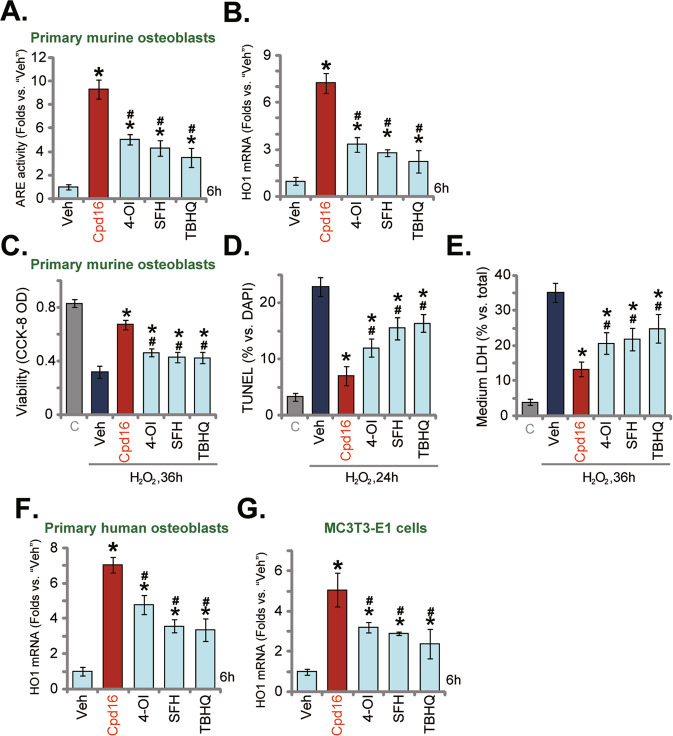
Cpd16-induced Nrf2 activation and osteoblasts protection against H_2_O_2_ were stronger than other known Nrf2 activators. The primary murine osteoblasts (**A**, **B**), human osteoblasts (**F**), and MC3T3-E1 murine osteoblastic cells (**G**) were treated with 25 μM of Cpd16, Sulforaphane (SFH), 4-octyl itaconate (4-OI), tert-butylhydroquinone (TBHQ), and cultured for 6 h, the relative ARE activity (**A**) and *HO1* mRNA (**B**, **F**, **G**) expression were tested. The primary murine osteoblasts were pretreated with 25 μM of Cpd16, SFH, 4-OI, or TBHQ for 2 h, followed by H_2_O_2_ (400 μM) stimulation, and viability, apoptosis, and necrosis were measured through CCK-8 (**C**), TUNEL-nuclei staining (**D**), and LDH releasing (**E**) assays, respectively. *P < 0.05 *versus* “Veh”. ^#^*P* < 0.05. *versus* “Cpd16”.

The activity of Cpd16 was compared to other known Nrf2 activators, including 4-OI [10, 44, 45], SFH [46, 47], and TBHQ [48, 49]. At shown in the primary murine osteoblasts, at the same concentration (25 μM), Cpd16-induced increases in ARE activity (Fig. 4A) and *HO1* expression (Fig. 4B) were significantly more potent than these other Nrf2 activators (4-OI, SFH, and TBHQ). Importantly, although each of the applied Nrf2 activators ameliorated H_2_O_2_-caused viability decreasing (Fig. 4C), apoptosis (Fig. 4D), and necrosis (LDH releasing, Fig. 4E) in primary murine osteoblasts. Cpd16-mediated osteoblast protection was more significant than these other activators (4-OI, SFH, and TBHQ) (Fig. 4C–E). In human osteoblasts and MC3T3-E1 murine osteoblastic cells, Cpd16-induced *HO1* mRNA expression was again more significant than the same concentration of 4-OI, SFH, or TBHQ (Fig. 4F, G). Therefore, Cpd16-induced Nrf2 activation and osteoblasts protection against H_2_O_2_ were stronger than other known Nrf2 activators.

### Cpd16 inhibits dexamethasone- and nicotine-induced oxidative injury in osteoblasts

**Fig. 5:**
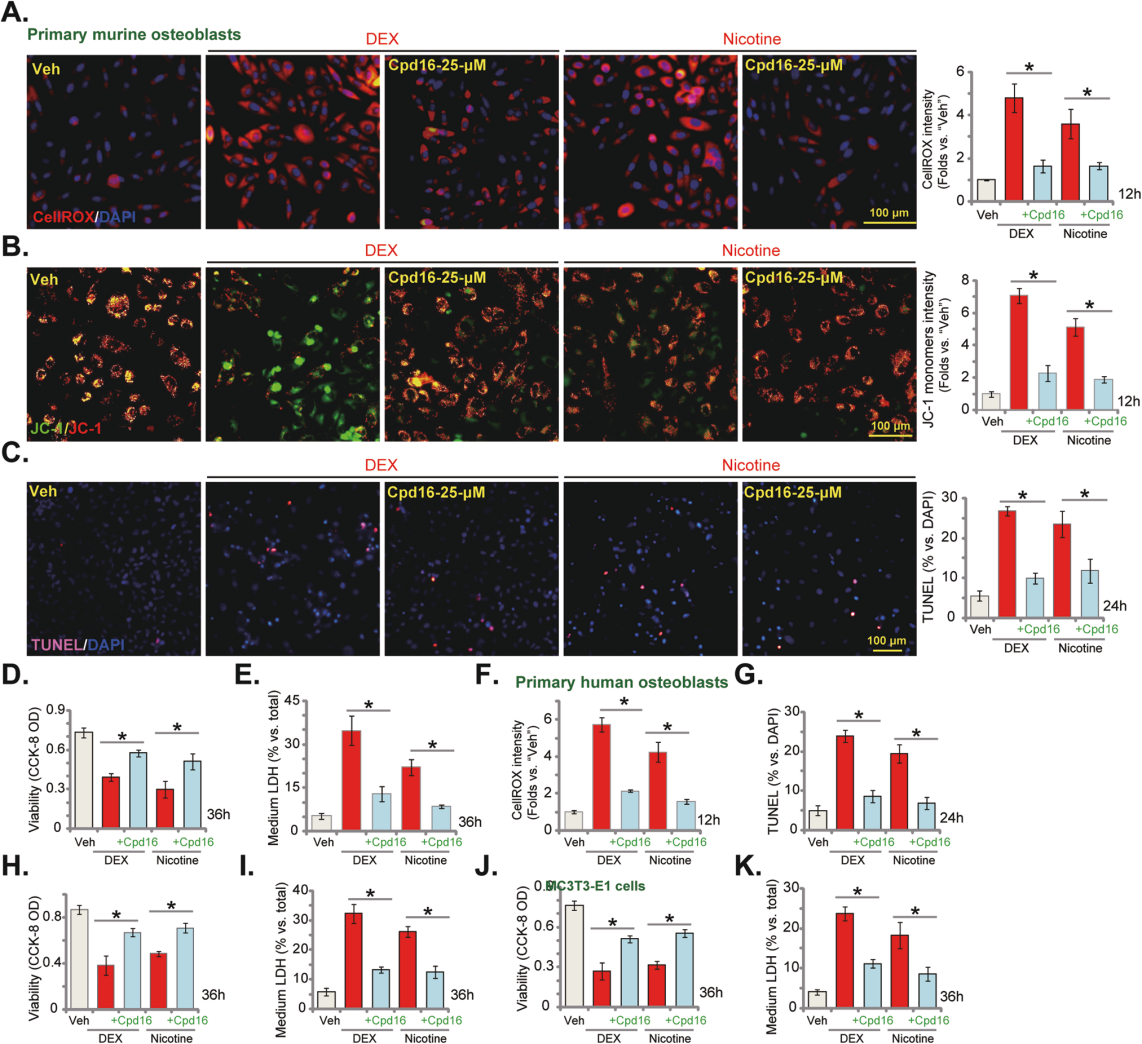
Cpd16 inhibits dexamethasone- and nicotine-induced oxidative injury in osteoblasts. The primary murine osteoblasts (**A**–**E**), human osteoblasts (**F**–**I**), or the MC3T3-E1 murine osteoblastic cells (**J**, **K**) were pretreated (for 2 h) with Cpd16 (25 μM), followed with or without dexamethasone (DEX, 2 μM) or nicotine (1 μM) treatments, ROS, depolarization of mitochondria, apoptosis, viability, and necrosis were tested by CellROX staining (**A**, **F**), JC-1 staining (**B**), TUNEL-nuclei staining (**C**, **G**), CCK-8 (**D**, **H**, **J**), and LDH releasing (**E**, **I**, **K**) assays, respectively. *P < 0.05. Scale bar = 100 μm (**A**–**C**).

Besides H_2_O_2_, other stimuli, including dexamethasone (DEX) [28, 29, 36, 50] and nicotine [30, 51–53], can also provoke oxidative injury in osteoblasts, which could be alleviated by Nrf2. As shown, DEX and nicotine both induced profound oxidative injury, increasing CellROX intensity (Fig. 5A) and JC-1 monomers (Fig. 5B) in murine osteoblasts. Importantly, Cpd16 largely inhibited DEX- and nicotine-induced oxidative stress in murine osteoblasts (Fig. 5A, B). Functional studies demonstrated that the novel Nrf2 activator significantly attenuated cytotoxicity by DEX and nicotine in murine osteoblasts. DEX- and nicotine-induced apoptosis (Fig. 5C), viability decreasing (Fig. 5D), and necrosis (LDH releasing, Fig. 5E) were largely inhibited by Cpd16. In human osteoblasts, DEX-/nicotine-induced oxidative stress (CellROX intensity assays, Fig. 5F), apoptosis (Fig. 5G), viability decreasing (Fig. 5H), and necrosis (Fig. 5I) were ameliorated by Cpd16. In MC3T3-E1 murine osteoblastic cells, Cpd16 treatment potently inhibited DEX- and nicotine-induced viability decreasing (Fig. 5J) and necrosis (Fig. 5K) as well. Thus, Cpd16 inhibited dexamethasone-/nicotine-induced oxidative injury in osteoblasts.

### Nrf2 activation is indispensable for Cpd16-mediated osteoblast cytoprotection

**Fig. 6:**
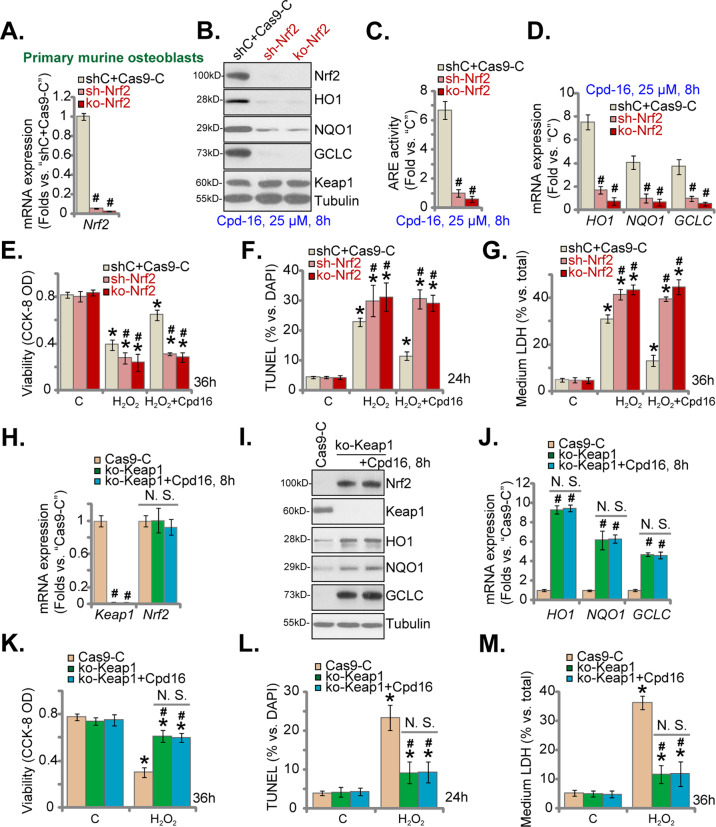
Nrf2 activation is indispensable for Cpd16-mediated osteoblast cytoprotection. *Nrf2* mRNA expression in the listed primary murine osteoblasts was measured (**A**); the osteoblasts were treated with Cpd16, and listed proteins (**B**), ARE activity (**C**), and listed mRNAs (**D**) were measured; the murine osteoblasts were pretreated with Cpd16 (25 μM) for 2 h, followed by H_2_O_2_ (400 μM) stimulation, with cell viability, apoptosis and necrosis tested by CCK-8 (**E**), TUNEL-nuclei staining (**F**), and LDH releasing **G** assays, respectively. *Keap1*-*Nrf2* mRNA expression in the ko-Keap1 or Cas9-C murine osteoblasts was measured (**H**); the ko-Keap1 murine osteoblasts were also treated with or without Cpd16 (25 μM), expression of listed proteins and mRNAs (**I**, **J**) was measured; the ko-Keap1 osteoblasts were pretreated with Cpd16 (25 μM) for 2 h, followed by H_2_O_2_ (400 μM) stimulation; cell viability, apoptosis, and necrosis were measured by CCK-8 (**K**), TUNEL-nuclei (**L**), and LDH releasing **M** assays, respectively. *P < 0.05 *versus* “C” cells. ^#^*P* < 0.05 *versus* “shC+Cas9-C”/“Cas9-C” osteoblasts. “N.S.” stands for the non-statistical difference (*P* > 0.05).

Genetic strategies were employed to silence Nrf2. Nrf2 shRNA-expressing lentivirus [10, 30] was added to cultured primary murine osteoblasts, and stable osteoblasts were formed after puromycin selection: namely “sh-Nrf2” osteoblasts. Moreover, Nrf2 knockout (KO) osteoblasts were established by transducing the CRISPR/Cas9-Nrf2-KO construct to the Cas9-expressing murine osteoblasts. After selection (through puromycin) and KO screening the single stable Nrf2 KO murine osteoblasts, namely “ko-Nrf2” osteoblasts, were formed. Control cells were with the scramble control shRNA (“shC”) plus the CRISPR/Cas9 empty vector (“Cas9-C”). As shown, *Nrf2* mRNA expression (Fig. 6A) and Cpd16 (25 μM, 8 h)-induced Nrf2 protein stabilization (Fig. 6B) were nullified by Nrf2 shRNA/KO in murine osteoblasts. Moreover, Cpd16 (25 μM, 8 h)-provoked ARE luciferase reporter activity increase (Fig. 6C), mRNA (Fig. 6D), and protein (Fig. 6B) expression of Nrf2-ARE response genes were almost completely reversed following Nrf2 silencing or KO.

In Nrf2-depleted osteoblasts H_2_O_2_-caused viability reduction (Fig. 6E), apoptosis (Fig. 6F), and necrosis (LDH releasing, Fig. 6G) were exacerbated, suggesting that the basal Nrf2 activation can attenuate H_2_O_2_-induced cytotoxicity. More importantly, Cpd16 was completely ineffective against H_2_O_2_ in Nrf2-depleted murine osteoblasts (Fig. 6E–G). Thus, Nrf2 depletion abolished Cpd16-induced cytoprotective activity in osteoblasts.

We further hypothesized that Cpd16 should be invalid in Nrf2 over-activated osteoblasts. Thus, a described CRISPR/Cas9-Keap1-KO construct [10, 30] was stably transduced to the Cas9-expressing osteoblasts to establish “ko-Keap1” osteoblasts, showing depleted *Keap1* (Fig. 6H, I). Keap1 KO did not affect *Nrf2* mRNA expression in murine osteoblasts (Fig. 6H), but induced robust Nrf2 protein stabilization (Fig. 6I). It also robustly increased expression of Nrf2 response genes (Fig. 6I, J). As a result, H_2_O_2_-induced viability decrease (Fig. 6K), apoptosis (Fig. 6L), and necrosis (LDH releasing, Fig. 6M) were largely attenuated by Keap1 KO. Significantly, in Keap1 KO osteoblasts, Cpd16 (25 μM) was unable to further enhance Nrf2 activation (Fig. 6I, J). Neither did it offer additional cytoprotection against H_2_O_2_ (Fig. 6K–M). In ko-Keap1 murine osteoblasts Cpd16 was unable to inhibit H_2_O_2_-induced viability decreasing (Fig. 6K), apoptosis (Fig. 6L), and medium LDH releasing (Fig. 6M). These results provided additional evidence to support that Nrf2 is indispensable for Cpd16-induced osteoblast cytoprotection.

### Keap1 Cys151S mutation abolishes Cpd16-induced Nrf2 cascade activation and osteoblasts protection

**Fig. 7:**
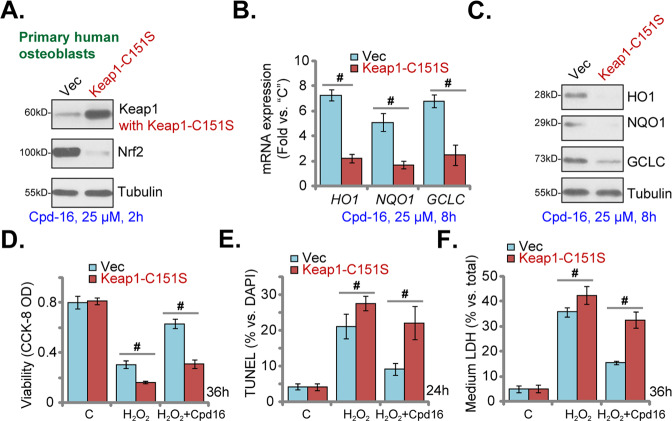
Keap1 Cys151S mutation abolishes Cpd16-induced Nrf2 cascade activation and osteoblasts cytoprotection. Stable primary human osteoblasts with Cys151S Keap1 (“Keap1-C151S”) or Vector were treated with Cpd16 (25 μM), listed proteins and genes were measured (**A**–**C**). Alternatively, the primary human osteoblasts were pretreated with Cpd16 (25 μM) for 2 h, followed by H_2_O_2_ (400 μM) stimulation, and cell viability, apoptosis, and necrosis were tested by CCK-8 (**D**), TUNEL-nuclei staining (**E**), and LDH releasing **F** assays, respectively. ^#^*P* < 0.05.

Cpd16 activated Nrf2 signaling by acting as the Keap1-Nrf2 protein–protein interaction inhibitor [54, 55]. However, the detailed mechanisms are still elusive. Therefore a Cys151S mutant Keap1 [56] vector was stably transduced to primary human osteoblasts. Keap1 (C151S) expression was confirmed in Fig. 7A. Importantly Cpd16-induced Nrf2 protein stabilization was largely inhibited by Keap1 mutation in human osteoblasts (Fig. 7A). Moreover, Cpd16-initiated expression of Nrf2-dependent genes was largely inhibited by the Keap1 mutation (Fig. 7B, C). As shown, H_2_O_2_-induced cell viability decreasing (Fig. 7D), apoptosis (Fig. 7E), and necrosis (Fig. 7F) were augmented in the Keap1-mutant human osteoblasts. Moreover, Cpd16-induced osteoblast cytoprotection against H_2_O_2_ was almost reversed in osteoblasts with the mutant Keap1 (Fig. 7D–F). These results implied that Keap1 cysteine (151) alkylatation could be vital for Cpd16-stimulated Nrf2 cascade activation, exerting osteoblast cytoprotection against H_2_O_2_.

### Cpd16 administration largely ameliorates trabecular bone loss in OVX mice

**Fig. 8:**
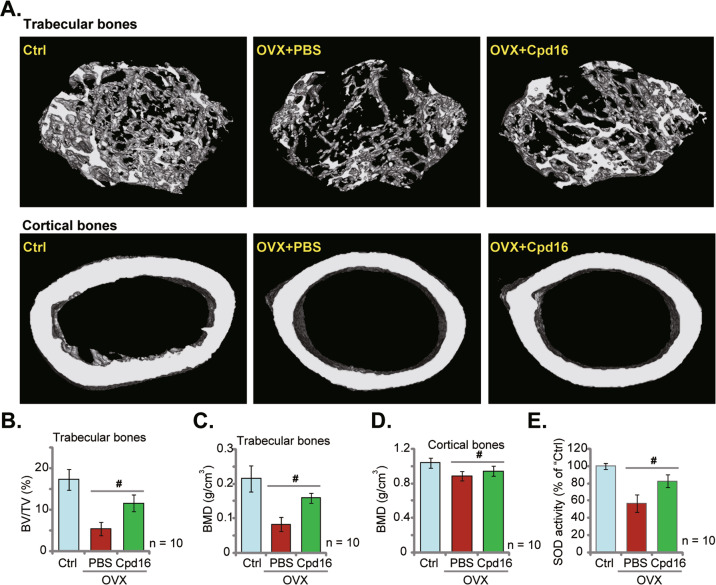
Cpd16 administration largely ameliorates trabecular bone loss in OVX mice. The female C57/BL6 mice were subject to bilateral ovariectomy (OVX) procedure. Afterward, Cpd16 (at 5 mg/kg) or PBS were intraperitoneally injected (i.p.) at the first day of each week, and mice were sacrificed after 8 weeks. The representative micro-CT images of trabecular bones and cortical bones were presented (**A**). BV/TV (%, **B**) and bone mineral density (BMD, g/cm^3^, **C**) of trabecular bones were calculated. BMD of cortical bones was recorded as well (**D**). The relative SOD activity in the left tibia bone tissues in different groups was shown (**E**). The control group mice were orally administered with PBS (“Ctrl”). 10 mice per group. ^#^*P* < 0.05.

To examine the potential activity by Cpd16 in vivo, the mouse OVX model was utilized. The representative micro-CT images demonstrated that weekly intraperitoneal injection of Cpd16 (5 mg/kg) largely ameliorated trabecular bone loss in the OVX mice (Fig. 8A). The reductions of BV/TV (%, Fig. 8B) and BMD (Fig. 8C) in trabecular bones of OVX mice were largely alleviated with Cpd16 administration. Moreover, BMD of the cortical bones was also slightly decreased eight weeks after OVX (Fig. 8D), which was also inhibited following Cpd16 administration (Fig. 8D). Whether the antioxidant mechanism was activated by Cpd16 in vivo was determined. As shown, the SOD activity in the left tibias was significantly decreased in OVX group mice (8 weeks after OVX, Fig. 8E), while Cpd16 administration remarkably elevated it (Fig. 8E). These results showed that Cpd16 administration largely ameliorated oxidative injury and trabecular bone loss in OVX mice.

## Discussion

The transcription factor Nrf2 promotes the transcription and expression of a large number of antioxidant and/or defense genes, serving as a potential therapeutic target involved in the mitigation oxidative injury in osteoblasts [10, 13, 15, 16, 24, 28, 36]. Forced activation of Nrf2 signaling in osteoblasts/osteoblastic cells, using different agents or genetic strategies, was able to significantly inhibit oxidative injury by H_2_O_2_ and a number of other oxidative stimuli [10, 13, 15, 16, 24, 28, 36].

Here in different osteoblasts, Cpd16 treatment at only micro-molar concentrations induced disassociation of Keap1-Nrf2, stabilization of Nrf2 protein and following nuclear translocation, and enhanced ARE reporter activity as well as transcription of Nrf2 response genes (*HO1*, *GCLC*, and *NQO1*) in cultured osteoblasts/osteoblastic cells. Significantly, Cpd16 ameliorated oxidative injury in H_2_O_2_-stimulated osteoblasts.

We found that Cpd16-induced Nrf2 activation and osteoblasts protection against H_2_O_2_ were stronger than other known Nrf2 activators (SFH, 4-OI, and TBHQ). One possibility is that Cpd16 could induce Keap1 cysteine (151) alkylatation, leading to dramatic Keap1-Nrf2 disassociation and direct Nrf2 cascade activation. Indeed, we found that Keap1 Cys151S mutation abolished Cpd16-induced Nrf2 cascade activation and osteoblasts protection in primary human osteoblasts. The detailed mechanisms warrant further characterizations.

H_2_O_2_ and other oxidative stimuli (i.e. DEX), while activating cell apoptosis, can simultaneously induce programmed necrosis [10, 30, 57, 58]. The latter is a mitochondria-dependent active and programmed cell necrosis cascade [10, 42, 59]. Oxidative stimuli can induce p53’s translocation to mitochondria and form a multiple-protein complex with CyPD and ANT-1 [10, 42, 59]. The formation of the complex will thereafter induce mPTP open, mitochondrial depolarization, and cell necrosis [10, 42, 59]. Here, Cpd16 potently suppressed H_2_O_2_-stimulated programmed necrosis. H_2_O_2_-induced mitochondrial association of p53-CyPD-ANT1, depolarization of mitochondria, and cell necrosis were largely inhibited by Cpd16 pretreatment.

DEX can directly induce oxidative injury and osteoblast cell death, and it is a key factor for the progression of osteoporosis and osteonecrosis [60], which can be inhibited by Nrf2 activation [29, 50, 61]. Here DEX-caused oxidative injury and death in osteoblasts were largely attenuated by Cpd16. This novel Nrf2 small molecule activator should have promising value for the treatment of DEX-related bone injuries.

Sustained and/or high-dose nicotine exposure can significantly inhibit cell proliferation and differentiation in osteoblasts, and inhibit alkaline phosphatase (ALP) activity and collagen synthesis [51–53]. These changes together will eventually induce apoptosis, serving as the primary mechanism of cigarette smoke-related osteoporosis [51–53]. In the primary rat osteoblasts, nicotine was shown to inhibit multiple osteogenic and angiogenic genes [53]. We found that treatment with Cpd16 potently inhibited nicotine-induced oxidative injury and death of osteoblasts.

Osteoporosis seriously affects the life of the elderly people, especially postmenopausal women [62, 63]. One key pathophysiological feature of osteoporosis is osteoblast dysfunction, resulting in decreased bone formation [62, 63]. Oxidative stress-induced dysfunction and death of osteoblasts is the primary reason for the bone loss during the development of osteoporosis [64, 65]. Therefore, reducing oxidative stress, i.e. using Nrf2 activators, can protect osteoblasts and inhibit their death, which has a promising effect on improving osteoporosis [64, 65]. Here, Cpd16 inhibited H_2_O_2_-caused oxidative injury and death in cultured osteoblasts. The Nrf2 activator also largely ameliorated oxidative stress and trabecular bone loss in OVX mice. Therefore, it should have promising value for osteoporosis management.

## Materials and methods

### Reagents

Cpd16 was synthesized by Shanghai Ruilu Chemicals (Shanghai, China). Dexamethasone (DEX), nicotine, cycloheximide, Sulforaphane (SFH), 4-octyl itaconate (4-OI), tert-butylhydroquinone (TBHQ), MG-132, and hydrogen peroxide (H_2_O_2_) were purchased from Sigma (St Louis, MO). Antibodies were described early [30].

### Culture of primary murine/human osteoblasts and MC3T3-E1 murine osteoblastic cells

As described previously [10, 30], the trabecular bone fragments of written informed consent healthy donors were minced, washed, and digested. Thereafter, the primary human osteoblasts were obtained and cultivated in the described medium [30]. Medium was renewed twice a week. The primary murine osteoblasts were obtained and cultured as described [10, 30]. The established MC3T3-E1 cells were provided by Dr. Zhou and cultivated as reported [50]. The protocols were with approval from the Ethics Board of Shanghai Jiao Tong University School of Medicine.

### Genetic modifications in osteoblasts

For Nrf2 silencing, the lentiviral construct encoding short hairpin RNA (shRNA) sequence of Nrf2 [10, 30] was transduced to the primary murine osteoblasts. Following puromycin-mediated selection, the stable osteoblasts were formed. CRISPR/Cas9-induced knockout (KO) of Keap1 or Nrf2 as well as the establishment of the single stable osteoblasts were described previously [10, 30].

### Keap1 mutation

The GV248 lentiviral Cys151S mutant Keap1 construct (no GFP) was from Dr. Liu at Jiangsu University [66] and was stably transduced to the osteoblasts. Cys151S Keap1 was checked by western blotting.

Other assays, including cell viability CCK-8 assay, the Caspase-3/-9 activity, the JC-1 fluorescence testing mitochondrial depolarization, the CellROX fluorescence staining of ROS, the cell necrosis assay by measuring medium LDH contents, the lipid peroxidation by measuring the reactive substances (TBAR) activity, NQO1 activity assay, ARE reporter activity assay, and single strand DNA (ssDNA) ELISA as well as qRT-PCR, co-immunoprecipitation (Co-IP), western blotting, Annexin V flow cytometry and nuclear TUNEL staining assays were reported in the previous studies [10, 30]. Primers were provided by Dr. Jiang at Nanjing Medical University [67, 68]. The uncropped blotting images were presented in Figure S1.

### The murine ovariectomized (OVX) procedure and micro-CT analyses

The female C57/BL6 mice, at 7 weeks of age and 21–22 g of weight, were purchased from SLAC (Shanghai, China). Mice were anesthetized as described [69] and the detailed protocols of OVX were reported early [69]. Cpd16 (5 mg/kg) or PBS was intraperitoneally injected (i.p.) at the first day of each week. Micro-CT analyses were described in an early study [70]. In brief, the OVX mice and the control mice were scanned under the micro-CT equipment (Skyscan 1176, Belgium) 8 weeks after OVX procedure and the high-resolution scanogram were retrieved [70]. The dataset was reconstructed under a CT analyzer software and bone erosion was calculated using the in-house Fiji script [70]. The trabecular bone volume (BV) versus the total volume (TV), in %, was measured. The bone mineral density (BMD, g/cm^3^) of trabecular bones and cortical bones was calculated as well [70]. After completion of micro-CT, the right tibias of the mice were collected and superoxide dismutase (SOD) activity in the fresh bone tissues was analyzed by a SOD ELISA kit (Thermo-Fisher Invitrogen, Shanghai, China). All animal experiments were conducted under protocols approved by IACUC of Soochow University.

### Statistical analyses

Statistical analysis was described early [10, 30]. *P* < 0.05 was considered as a statistically significant difference. Quantified values were mean ± standard deviation (SD). All in vitro experiments were repeated five times and similar results were obtained.

## Supplementary information

Figure S1. The uncropped blotting images

## Data Availability

All data are available upon request.
