# Supplementary figures and images for: Nrf2 signaling activation by a small molecule activator compound 16 inhibits hydrogen peroxide-induced oxidative injury and death in osteoblasts
Source: Cell Death Discov. 2022 Aug 8;8:353. doi: 10.1038/s41420-022-01146-7 (PMC9360014; doi:10.1038/s41420-022-01146-7)

Figure S1.The uncropped blotting images of the study.

Figure 1.

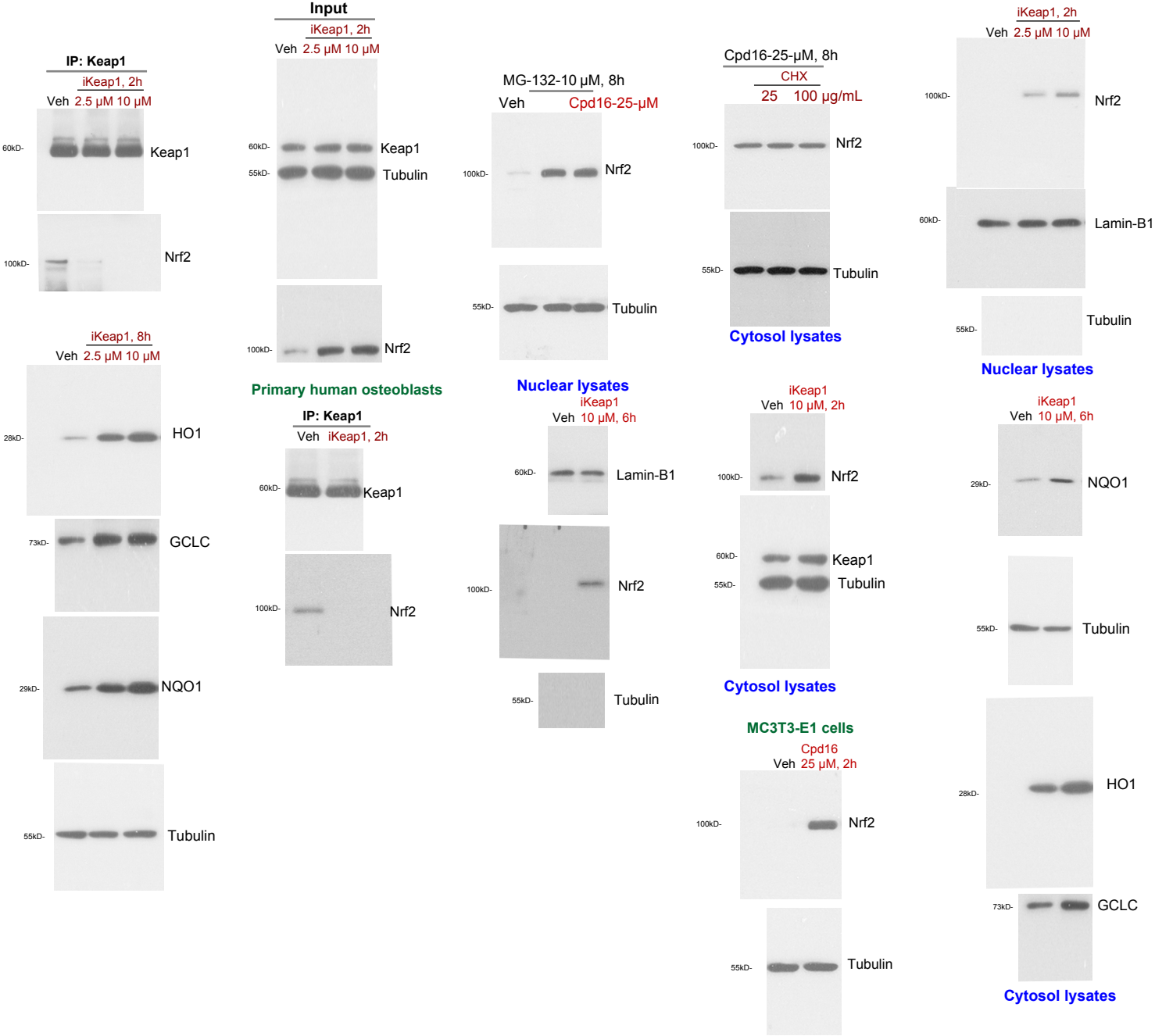

Figure 3.

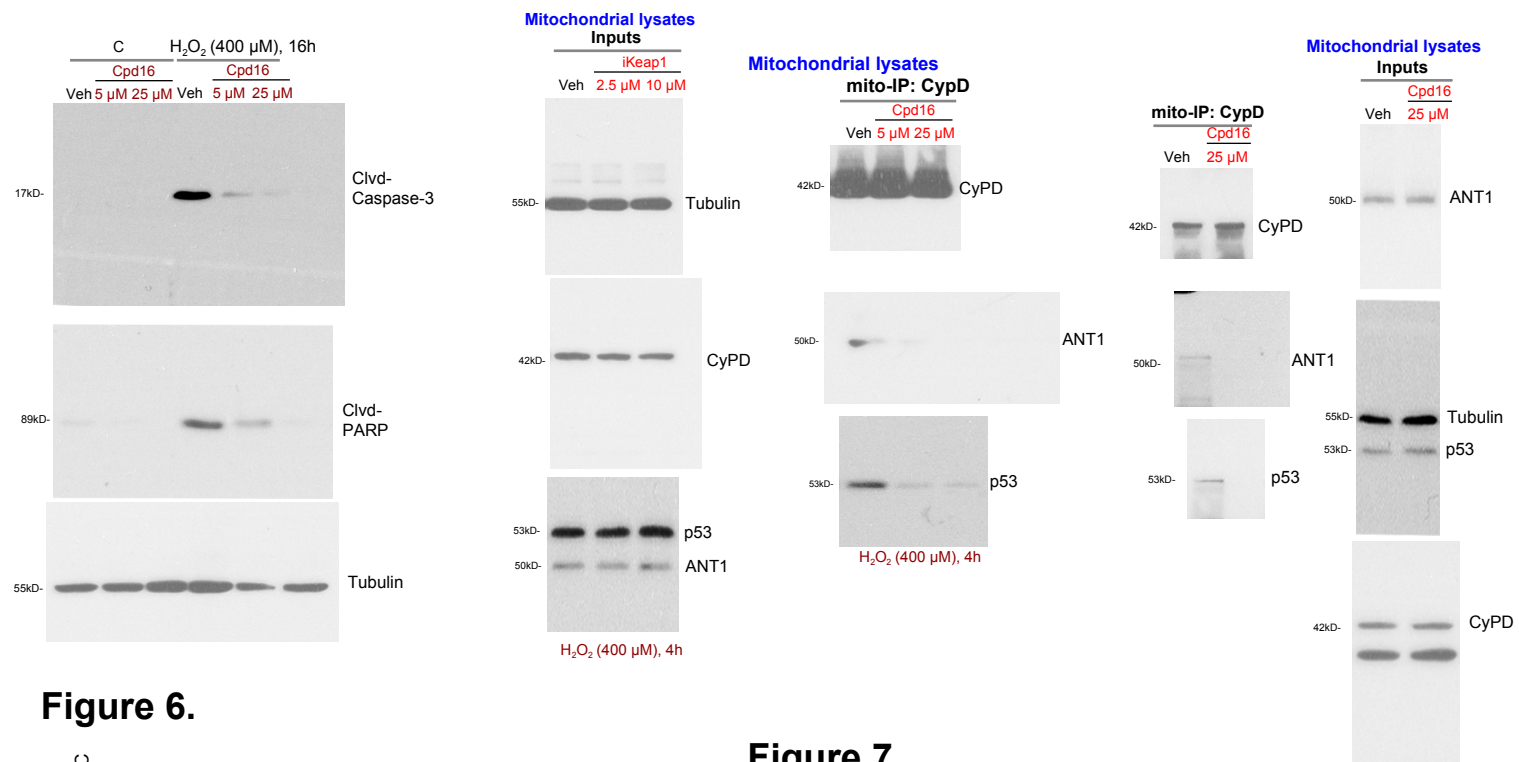

Figure 6.

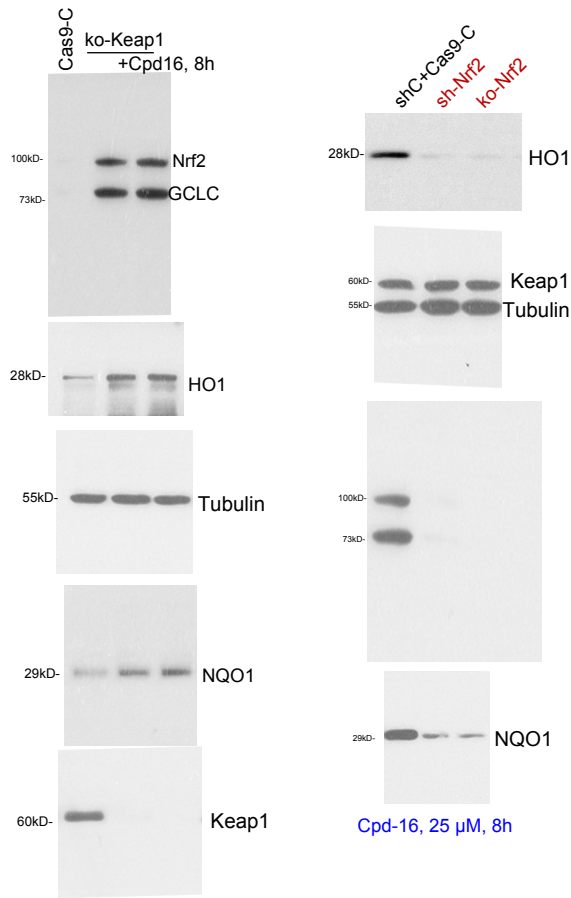

Figure 7.

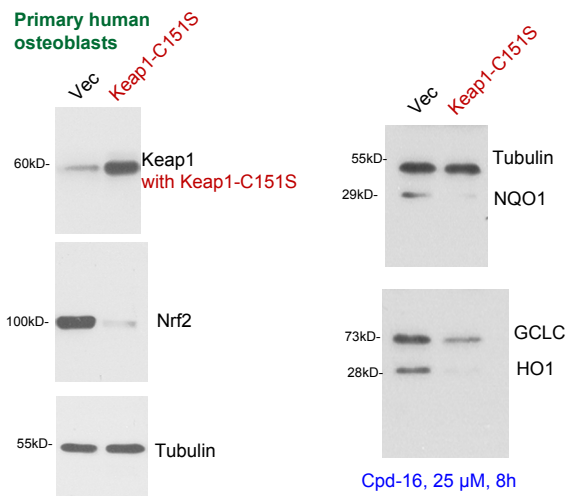

Supplement: Supplementary file 1 — Figure S1. The uncropped blotting images [file 41420_2022_1146_MOESM1_ESM.pdf]
